# Social Relations and Everyday Consumption Rituals: Barriers or Prerequisites for Sustainability Transformation?

**DOI:** 10.3389/fsoc.2021.723464

**Published:** 2021-08-24

**Authors:** Magnus Boström

**Affiliations:** School of Humanities, Education, and Social Sciences, Örebro University, Örebro, Sweden

**Keywords:** lifestyle, social interaction, consumer culture, sustainable consumption, overconsumption

## Abstract

Macro-institutional structures and consumerist culture force and urge people to reproduce unsustainable levels of consumption. A crucial role for sociology, the article argues, is to address theoretically and empirically the intersection between social relations and (over)consumption. The purpose with this article is to address how social relations are involved in both reproducing and challenging consumer culture. This is done by emphasizing the intersection of consumer culture and socially integrating everyday rituals and drawing on literature on both voluntary and involuntary (the pandemic) disruption of consumer practices. The Covid-19 pandemic brings unexpected opportunities to highlight this intersection, as the pandemic offers a window of opportunity for lifestyle change. The review shows there are important lessons about both challenges and opportunities, gained from both voluntary and involuntary disruption of consumer practices.

## Introduction

The world faces escalating climate and ecological crises. As one response, there is a growing international appreciation of the need for systemic change of social and institutional forces that perpetuate contemporary consumerist lifestyles (Cohen 2020). Yet, societies generally continue to force and urge people to reproduce cultures and habits with unsustainable levels of consumption. There is an interplay of macro-institutional structures, cultural values, and social mechanisms behind patterns of mass and excess consumption (Boström 2020). This perspective article focuses on the specific role of social relations, which is often overlooked in both policy and research about (un)sustainable lifestyles and consumption. This topic cannot remain neglected if societies will have a chance to transform in sustainable directions. Sociology has an important and promising role in highlighting and studying the intersection between social relations and (over)consumption. It is argued here that this intersection constitutes a key barrier to sustainability directed lifestyle change. Our very basic need for social belonging and to maintain social relations is a key factor behind our urge to overconsume. At the same time, social support and healthy social relations are key for the sustainability transition, hence preconditions for change. Societies could encourage development of less consumerist ways of facilitating social relations, which would much likely also be beneficial for these relations (Kasser 2017). This perspectice article explores this ambiguity of social relations—as both barriers and preconditions in relation to consumption/lifestyle change—both theoretically and by reference to recent studies on both voluntary (e.g., volunary simplicity) and involuntary (Covid-19) disruption of consumer practices. Indeed, such living experiments (disruption of consumer practices) have the potential to reveal relations and patterns in everyday life, which otherwise are taken for granted. For instance, the Covid-19 pandemic brings unexpected opportunities to highlight this intersection between social relations and (over)consumption. The pandemic has triggered much discussion whether and how it offers a window of opportunity for transformative sustainability change, not the least for lifestyle change (Cohen 2020; De Haas et al., 2020). This article aims to address how social relations are involved in both reproducing and challenging consumer culture, particularly by emphasising a micro-sociological perspective on consumer culture and socially integrating everyday rituals. After introducing the theoretical perspective in the next section, the discussion is followed by reviewing studies that explore challenges and lessons gained from both voluntary and involuntary (the pandemic) disruption of consumer practices. The review takes the form of an integrative literature review (Snyder 2019), which is more selective than a systematic literature review, and aims to synthesize literature on a research topic to allow new perspectives to emerge.

## Everyday Rituals, Social Relations, and Consumption

A classic insight from social psychology is that our sense of meaning in life are fundamentally shaped by one’s social relations, both intimate such and more distant reference groups. By processes of socialization, people naturalize their everyday and social lives in their material contexts by developing worldviews, norms, roles, habits, desires, and identities (Berger and Luckmann 1966; Blumer 1969; Ghisleni 2017; Boström 2020). In contemporary societies consumer culture plays a key role in this socialization: people are born into social lives deeply shaped by mass consumption structures and forces (Schor 2005; Sassatelli, 2007). The consumer desires that are developed in the process are not just personal, they are essentially social (Belk et al., 2003).

Rook (1985) argued decades ago that consumer research largely has failed to recognize the strong link between consumer and ritual behaviour. I believe this claim is still very much valid. The sociologist Randal Collins (2005) developed a useful micro-sociological theory by combining insights from Emile Durkheim and Erving Goffman. Collins stressed the importance of interaction ritual chains for social integration and the maintenance of social relations. For Rook and Collins, rituals refer to a much broader phenomenon than formal ceremonies. Indeed, we engage in numerous rituals every day. Examples include conversations, having dinners together, body- and haircare, protest demonstrations, watching concerts or football games, playing games, shopping—all kinds of activities that groups of people are doing together, often in a habitualized manner. Participants may “play roles” and follow rules and scripts, with the result that rituals appear as relatively fixed episodic sequences (Rook 1985). For Collins, participation in interaction ritual chains cause positive emotional energies and bolster social solidarity. They are the glue of social life.

According to Collins, rituals include four ingredients—group assembly, barriers to outsiders, mutual focus of attention, shared mood—and lead to four types of outcomes: group solidarity, emotional energy in individuals, symbols of social relationship (sacred objects), and standards of morality (social norms). People coming together and paying mutual attention to something may feel the “electricity”—Durkheim used the word collective effervescence—involved in experiencing, doing, and desiring the same thing as the other group members. Because of the positive emotions and sense of solidarity, they repeat the rituals over and over again. Even if Collins stressed the importance of co-presence and concrete relations, more distant and abstract categories of belonging such as an idea or imagined community (nation, football team, brand community) are also important. Moreover, the growth of social media, online communities, and not the least the Covid-19 pandemic has showed the increase of digitalized interaction ritual. Symbols of relationships (sacred objects) remind of situations of heightened intersubjectivity: souvenirs, flags, logos, art, role models, artefacts, slogans, and so on. Symbols reify and prolong the shared experience contained in the ritual and makes it thing-like. Members of rituals feel morally right when they are exercising the ritual. Therefore, norms around rituals are established. Symbols must be respected. A ritual violated cause feelings of shame and moral uneasiness.

In contemporary consumer society, interaction rituals and consumption are tightly related (Spaargaren 2011). First, consumption provide for the shared focus of attention. Social groups gather and indulge together around physical objects: home furniture, mobile phones, fashion items, motor vehicles. It could also be a joint activity such as sharing food, giving presents, going out for a dinner, shopping together, watching a concert, go to an amusement park or travelling abroad for the weekend holiday. Feelings of connectedness or synchronicity during consumption act can intensify people’s enjoyment of an experience (Ramanathan and McGill 2007). The ritual may be emotionally energizing and socially integrating both *before* (jointly daydreaming, wishing, imagining, and planning), *during* (shopping, enjoying the commoditized activity), and *after* (accommodating it at home, using it, remembering) the purchase. Passionate longing for goods (houses, cars, luxury items) and services (vacation trips) may be what unites the family.

Second, consumer objects become material manifestations of the rituals, the “sacred” objects representing the rituals (e.g., a branded shoe) (Firat et al., 2013). An object that one is procuring—extravagant or mundane—can represent a bridge to another person or to a collective that one is affiliated with (Miller, 1998; Jenkins et al., 2011). In his books, *A theory of shopping* (1998) Miller builds on ritual theory to emphasize how love and care for others is a basic motivation behind much of ordinary shopping. Shoppers develop and imagine those social relationship which they care most about through the medium of selecting any kinds of goods, and not just the most symbolically laden goods such as cars and fashion.

Third, consumer objects provide for the scene or needed requisites: material resources that facilitate the ritual. A conversation may be facilitated by going out for a lunch. Sometimes considerable material expenses are needed for taking part in interaction ritual chains (Collins 2005). Material goods can constitute important part of an experience and imagination even when they are not the key focus of consumers’ attention (Jenkins et al., 2011).

The social drivers implicated above are also seen among lone consumers. They may find engagement and some human-like companionship by participation in gaming or brand communities or similar (Wang et al., 2021). For some, branded goods can be fetishized and appear as life partners. Segments of lone consumers may adopt strikingly materialist lifestyles and see products as a replacement of interpersonal relationships.

As we engage in everyday rituals that involve lots of consumption, for the pursuit of emotional energies and bolstering social bonds, and for expressing love, romance, and care, these social mechanisms provide a fertile ground for market expansion and exploitation, for commoditization and commercialization of intimate social life (Hochschild 2011; Brook et al., 2013). Even if—or rather, because—social relations take the central stage—such as the love couple’s romantic weekend trip by air to an exciting city in a foreign country—considerable amounts of material goods and resources are implicated.

## Voluntary Reduced Consumption

Previous studies of voluntary reduced consumption reveal both opportunities and difficulties with regards to how social relations shape conditions for the aspirations. An often-stated benefit with voluntary simplicity is that people get time for each other because they work fewer hours (Osikominu and Bocken, 2020; Rebouças and Soares, 2020). There are many creative, time-intensive, and less commoditized ways to foster social relations: family games, slow tourism, joint hobbies such as gardening, and so on. At the same time, people experimenting with anti-consumption strategies face challenges, and the most critical relates to the need to maintain and confirm social relations as well as socializing together (Grauerholz and Bubriski-McKenzie., 2012; Armstrong et al., 2016; Callmer 2019). There are conflicts among couples, family members and relatives and there can be lost friends (Cherrier et al., 2012; Osikominu and Bocken 2020). Moreover, anxieties that relate to social judgment and comparisons connected with status consumption, being reachable and mobile (by mobile phone and flying), and risks of not reaching the minimal level of socially required consumption constitute key social barriers even for committed downsizers (Isenhour 2010; Cherrier et al., 2012; Callmer 2019).

A particular difficulty in the family context is children’s peer pressure. Even voluntary simplifiers “must cross personal moral boundaries that they would not have to cross if they were not parents” and they “have to demonstrate their care and love for a child by consuming or showing proof of consumption,” (Walther and Sandlin., 2013 p. 43). Everyday rituals surrounding gift-giving and celebrations such as birthday parties can be particularly difficult to avoid (Hochschild 2011; Lorenzen 2017; Callmer 2019). Even environmentally conscious parents with critical views of consumer culture will find it hard to fully resist the overall materialist frame when they celebrate their children: “They both embrace and critique it, enjoy and seek to resist the emphasis on consuming things and experiences.” (Schoonmaker 2006 p. 232).

Shoonmaker’s study of birthday parties also revealed much creativity in developing alternative family rituals by making them less commercial and more focused on joint activities and spending time together. It should be stressed that children can also be drivers of change and invent new family rituals or modifying existing ones, such as pushing for vegan or vegetarian meals (Callmer 2019). Social support is crucial: Osikominu and Bocken (2020) found, in their interview study of voluntary simplifiers, that the most frequently mentioned “enablers” were the partner or new peers who think in similar ways. It is important also to emphasize the frequent observation around gains in quality of life, which stem from less materialist aspirations, more focus on social relations, as well as the development of creativity and DIY-skills (Kasser 2017, Hagbert and Bradley 2017; Callmer 2019, Osikominu and Bocken 2020; Rebouças and Soares 2020). Could similar positive experiences be gained even in cases of involuntary disruption of several consumer practices?

## Involuntary Reduced Consumption: The Effect of Covid-19

The Covid-19 pandemic has in several ways disrupted mainstream consumer culture, including high climate impact travelling. Whereas some kinds of consumption and consumer activities have been impossible or greatly restricted (e.g., travel, shopping, fashion), others have continued as before or accelerated (online shopping). The interesting question is if there is any long-term transformative potential embedded in the new experiences, particularly considering the intersection between social relations and consumption. To begin with, what many people desire most during the pandemic is not physical stuff, but activities such as socializing with relatives and friends (De Haas et al., 2020). People also long for distant travelling, an activity that people often do for spending time together in an emotionally energizing way. There has been a drastic decline in demand related to the hospitality industry (Jones and Comfort 2020).

Some of the reviewed literature on consumption, socializing and pandemic experiences observe that the pandemic has forced people to invent new ways (everyday rituals) to nurture social relations. People have learned to socialize *via* online platforms (Echegaray 2021), by “quaranteaming” and inventing virtual gatherings such as virtual dinner parties, religious services, weddings and music performances (Kirk and Rifkin 2020). Such embracing of technology has enabled reunion with distant families and friends (Sheth 2020). One can ask, hence, if physical co-presence is so important for creating positive emotions and maintaining social bonds as Collins (2005) suggested? Rapid digitalization is arguably changing the phenomenology of social order and human sociality (Ghisleni 2017). Still, zoom fatigue arises as well as demand for physical touch. Digital interaction rituals may be insufficient in the long-term for fostering healthy social relations (De Haas et al., 2020). Whereas digitalization (and new technological equipment) facilitate sustained social lives during the pandemic, it will not replace the need for physical and more emotionally energizing ways of maintaining social relations. It is nonetheless an open question if digital socializing can, to some extent, replace the need of resource-demanding travel, and in this way contribute to reducing ecological and climate footprints.

There is, arguably, more transformative potential (in terms of reduced consumption) contained in new everyday rituals that people invented as an alternative to shopping or travelling. Studies show how the pandemic brings new family practices at home. There are indications of more conversations, feelings of care and empathy, and appreciation of time with family (Badrkhani 2020; Dwari 2020). More meals at home imply more family interaction around food practices, including preparing and eating together: “eating with family and cooking turned into new entertaining activities,” (Ben Hassen et al., 2020 p.13). New activities include jointly engaging in baking, cooking, gardening, jigsaw puzzling, family games, joint walks in nature and urban parks, local tourism (Benjamin et al., 2020; Borsellino et al., 2020; Kirk and Rifkin 2020; Sofo and Sofo 2020; Echegaray 2021). A rise in demand for pet adoptions is observed (Kirk and Rifkin 2020), which can be a measure done to cope with feelings of loneliness. All these activities offer some possibilities to link the bonding among families and friends to less consumerist and resource-intensive rituals. However, it is important to bear in mind that family members spending more time together in the home are not necessarily interacting more or fostering healthy social relations. They may spend more time on internet with distant, superficial interaction, and topics such as overcrowded home environments and domestic violence are important to consider as well (De Groot and Lemanski 2020).

Difficulties to socialize during the pandemic may have resulted in a decline of the strength of other social mechanisms connected to excess consumption, such as status consumption (conspicuous consumption) (Cohen 2020) and fear of social judgment (Esposti et al., 2021). For some groups of young consumers, beauty and body care products as well as apparel was among the product categories associated most superfluous during this period (Esposti et al., 2021)

Constraint cause creativity. From the literature, there are indications of some similar learning experiences with involuntary disruption as with those of voluntary downsizing. New practices trigger improvisation and development of new Do-It-Yourself competences, for instance in relation to cooking, baking, gardening (Borsellino et al., 2020; Amicarelli and Bux 2021), maintenance, repurposing and repairing, (Ehgartner and Boons, 2020), digital technology (Badrkhani 2020; Bin et al., 2021; Perkins et al., 2021), and physical activity (Zwanka and Buff 2020). Bolstered feelings of DIY-competence as well as discovery of talent (cooking, playing music, etc.) can improve well-being (Ehgartner and Boons., 2020; Kirk and Rifkin 2020; Sheth 2020), hence being emotionally energizing and thus something people want to continue exercising. Well-being is good foundation to maintain practices.

## Concluding Discussion

People may be aware and highly critical of the dominance of an ecologically unsustainable consumerist culture and yet unable to resist it. A key reason is that social relations are too important to them and that they feel a need to continuously confirm the relations by exercising consumerist rituals. A full retreat from mass consumption culture may appear impossible as such would imply a failure to comply with deeply internalized social norms, habits, and expectations. Both positive and negative emotions are involved. On the one hand, consumerist everyday rituals are emotionally energizing, and reflect expressions of love, care, and friendship. On the other, lots of anxieties are involved by the fear of social judgement and risk of violating consumerist standards and norms. These are felt compelling, particularly if children are involved. Intimate social relations are therefore a highly potent fuel for consumption. Therefore, social relations constitute key barriers to lifestyle change and sustainability transformations. At the very same time, social relations are preconditions for change. People cannot confront the barriers alone; they need to support each other in finding alternatives and jointly question how social relational aspirations and pressures permeate consumption motivations and patterns. Important lessons can be gained from both voluntary and involuntary disruption of consumer practices. Both kinds of disruptions can be an eye-opener to people that relations are more important than stuff. Both show that people are adaptive and have capacities to creatively invent less consumerist and resource-demanding ways of doing stimulating things together.

To conclude, it is essential to keep a firm analytical focus on the sphere of social relations and everyday rituals, both physical and digital, in all efforts to transform societies towards sustainability and reduced consumption. Both negative and positive experiences of downsizing must be identified and understood, as well as conflicting forces. On the one hand, various institutional and social powers (Boström 2020; Cohen 2020) will seek to force people back to the “normality” of mass consumption habits after the pandemic. On the other hand, new experiences and rituals have had considerable time to morph into enduring habits. Sociology has an imperative role and responsibility to explore these conflicts, how less consumerist-oriented and more meaningful human relations can be established, and opportunities for transformative change, theoretically and empirically.

## Data Availability

The original contributions presented in the study are included in the article/supplementary material, further inquiries can be directed to the corresponding author.
